# Analysis of the Dental Caries Epidemiological Profile in Children of Benguela city, Angola

**DOI:** 10.3290/j.ohpd.b2805501

**Published:** 2022-03-14

**Authors:** Marcial António Simão Songa, Nemre Adas Saliba, Tânia Adas Saliba, Fernando Yamamoto Chiba, Suzely Adas Saliba Moimaz

**Affiliations:** a Master in Preventive and Social Dentistry, Full Professor, Department of Preventive and Restorative Dentistry, São Paulo State University (UNESP), School of Dentistry, Araçatuba, São Paulo, Brazil. Experimental design, wrote the manuscript, proofread the manuscript, and contributed substantially to discussion.; b Ph.D. in Preventive and Social Dentistry, Full Professor, Department of Preventive and Restorative Dentistry, São Paulo State University (UNESP), School of Dentistry, Araçatuba, São Paulo, Brazil. Idea, hypothesis, experimental design, wrote the manuscript, proofread the manuscript, performed statistical evaluation, and contributed substantially to discussion.; c Ph.D. in Legal Dentistry and Deontology, Associate Professor, Department of Preventive and Restorative Dentistry, São Paulo State University (UNESP), School of Dentistry, Araçatuba, São Paulo, Brazil. Experimental design, wrote the manuscript, proofread the manuscript, performed statistical evaluation, and contributed substantially to discussion.; d Ph.D. in Preventive and Social Dentistry, Assistant Professor, Department of Preventive and Restorative Dentistry, São Paulo State University (UNESP), School of Dentistry, Araçatuba, São Paulo, Brazil. Experimental design, wrote the manuscript, proofread the manuscript, performed statistical evaluation, and contributed substantially to discussion.; e Ph.D. in Preventive and Social Dentistry, Full Professor, Department of Preventive and Restorative Dentistry, São Paulo State University (UNESP), School of Dentistry, Araçatuba, São Paulo, Brazil. Idea, hypothesis, experimental design, wrote the manuscript, proofread the manuscript, performed statistical evaluation, and contributed substantially to discussion.

**Keywords:** dental caries, child, Angola, health services accessibility

## Abstract

**Purpose:**

To analyse the epidemiological profile of dental caries in children aged 5 and 12 years in the city of Benguela, Angola.

**Materials and Methods:**

This was an observational, analytical, cross-sectional study conducted in 2019 with 190 12-year-old schoolchildren and 240 5-year-old schoolchildren from the public education system in Benguela, Angola. The relationship between dental caries and dental characteristics, sociodemographic factors, access to dental services, oral hygiene practices, and eating habits was analysed. Dental condition was evaluated using the dmft and decayed, missing, and filled teeth (DMFT) indices.

**Results:**

It was found that 62.63% (n = 119) of 12-year-old students and 42.08% (n = 101) of 5-year-old students were free from dental caries. The average DMFT was 0.76 + 1.35 and dmft was 2.19 + 2.95. The majority of children (56.51%) had never been to the dentist, had no dental elements restored, and none of the students used dental floss. The proportion of students who consumed sweets every day was higher at 5 years of age (46.25%) than at 12 years of age (22.63%). There was a statistically significant association (P = 0.01) between the higher incidence of dental caries and peri-urban location among 5-year-old schoolchildren.

**Conclusion:**

This study showed that the prevalence of dental caries in the permanent dentition of schoolchildren in Benguela is very low; however, the situation is critical in the primary dentition, especially in the peri-urban area. The limited access to dental surgeons and lack of treatment for affected teeth highlight the need to implement and develop public policies to promote oral health.

Dental caries is the most common oral health problem, and its prevention, treatment, and rehabilitation are major concerns for healthcare systems worldwide.^24^ Public health concerns associated with oral diseases cause statistically significant economic and health damage to the population, significantly reducing the quality of life of those affected.^22^ In this context, epidemiological studies on oral health conditions are fundamental because they allow for the collection of data on the prevalence and severity of oral diseases in different populations; provision of subsidies for the planning of interventions for health promotion, prevention, and rehabilitation; and evaluation of the outcomes of the implemented health strategies and policies.^29^ According to the World Health Organization’s Global Oral Health Database, the global prevalence of dental caries in schoolchildren is estimated to be 60–90%, with an increasing incidence in low- and middle-income countries, reinforcing the importance of studies to monitor this condition, particularly in populations with poor socioeconomic conditions and limited access to healthcare and prevention services.^30^ Furthermore, in countries with high rates of dental caries in the primary dentition, early childhood caries is becoming a statistically significant health problem. This disease pattern may be related to the process of transition to the modern diet and adoption of unhealthy lifestyles, noting that decayed primary teeth are often not treated, resulting in a negative impact on general health, quality of life, growth, and development of children, including a negative impact on school performance and attendance.^25,30^

A recent systematic review showed that the prevalence of dental caries in children living in the Middle East and North Africa region is high and is related to behavioural, cultural, and socioeconomic factors such as family background, oral hygiene practices, and eating habits, highlighting the importance of identifying and understanding these variables as tools for disease control and prevention.^9^ In the World Health Organization’s African Region, where 80% of the population has a low socioeconomic status, oral diseases negatively impact the health and quality of life of the people. The majority of this population has limited access to appropriate oral health services because of the uneven distribution of health professionals and a lack of adequate and functional infrastructure in the primary healthcare system, resulting in a high prevalence of oral diseases, a need for treatments, and demand for basic oral healthcare services, posing major challenges for healthcare systems in the region.^31^ As an aggravating factor, there is a scarcity of population-based data on oral health status in African countries, despite the evidence that oral health problems are one of the ten main reasons for outpatient care in the sub-Saharan African region.^17^

Considering this alarming lack of information, the aim of this study was to analyse the epidemiological profile of dental caries, oral hygiene practices, and eating habits in schoolchildren aged 5 and 12 years in the city of Benguela, Benguela Province, Angola. To date, there has been no scientific article on the oral health conditions of the population in the province of Benguela; hence, this research will serve as a reference point for interventions in the area of oral health, at the provincial level of Benguela and central and southern Angola.

## Materials and Methods

This is an observational, analytical, and cross-sectional epidemiological study conducted in 2019 with children aged 5 and 12 years in public schools in Benguela, Angola. The study included students aged 5 and 12 years, of both genders, who were regularly enrolled in public schools in the city of Benguela, were residents of the city, and whose legal guardians signed a free and informed consent form. Schoolchildren with neuropsychomotor disabilities who refused clinical examination, had physical limitations that made oral clinical examination impossible, and were absent after five data collection attempts were excluded from the study.

The list of all public primary schools based in the city of Benguela was obtained through a meeting with the head of the Municipal Education Department of Benguela. Based on this information, the school principals were contacted to obtain the nominal list of all children aged 5 and 12 years who were regularly enrolled. The entire study consisted of all students aged 5 and 12 years, who were enrolled in 50 schools of Benguela. In the first phase, a cluster sampling was performed to select 10 schools from the urban, peri-urban, and rural areas of the city, in proportion to the number of schools in the locality. After selecting the schools, the students were divided into two strata, 5- and 12-year-olds, in order to select the sample units. The sample size was calculated proportionally to each stratum, considering the population of children aged 5 and 12 years enrolled in the selected schools and a statistical significance level of 5%. The total sample size was determined in 430 schoolchildren, 240 of whom were aged 5 years and 190 were aged 12 years. Each student received an identification number, and proportional stratified random sampling was performed.

The dmft and DMFT indices were used to investigate the prevalence and severity of dental caries in primary and permanent dentition, using the codes and criteria for evaluating the dental condition recommended by the World Health Organization, as well as the manual for surveys in oral health.^29^ Data were collected by a single team, consisting of an examiner who had previously been trained and a recorder that was previously calibrated. On calculating the kappa coefficient, an intraexaminer agreement degree of 0.92 was obtained. The examinations were conducted in the school yards, in a well-ventilated place with natural light, using the WHO millimetre periodontal probe and a flat mouth mirror. A questionnaire was administered to the students’ legal guardians to obtain information regarding the participants’ sociodemographic characteristics, use of dental services, eating habits, and oral hygiene.

Data were analysed using descriptive statistical techniques, and the results are presented in the tables. The association between dental caries experience and sociodemographic characteristics, use of dental services, eating habits, and oral hygiene was analysed using the Chi-square test, and the odds ratio was calculated to determine the chances of occurrence of the disease. Data processing and analysis were performed using the Epi Info software version 7.2.2, adopting a statistical significance level of 5%.

The study was conducted in accordance with the ethical principles of the Declaration of Helsinki and approved by the Ethics and Research Committee of the Instituto Superior Politécnico de Benguela and by the Benguela Municipal Education Department. Authorisation for the participation of students in the research was obtained through an informed consent form signed by their legal guardians.

## Results

Of the 430 students enrolled in this study, 50.70% were males and 49.30% were females. As seen in Table 1, approximately half of them were free from dental caries experience (51.16%). The analysis of the strata showed that the prevalence of dental caries was higher in 5-year-old schoolchildren (57.92%) than in 12-year-old schoolchildren (37.37%). The average dmft index at 5 years was 2.19 + 2.95 (95% CI = 1.81–2.56), with minimum and maximum values of 0 and 15, respectively, while the average DMFT index at 12 years was of 0.76 + 1.35 (95% CI = 0.57–0.95), with minimum and maximum values of 0 and 7, respectively.

**Table 1:** Absolute and percentage distribution of schoolchildren aged 5 and 12 years, according to the dmft and DMFT indexes, respectively (Benguela, Angola, 2019)

dmft/DMFT	Age				Total	
	5-year-old		12-year-old			
	n	%	n	%	n	%
0	101	42.08	119	62.63	220	51.16
1	31	12.92	35	18.42	66	15.35
2	40	16.67	21	11.05	61	14.19
3	11	4.58	5	2.63	16	3.72
4	17	7.08	5	2.63	22	5.12
5	7	2.92	1	0.53	8	1.86
6	7	2.92	1	0.53	8	1.86
7	5	2.08	3	1.58	8	1.86
8	7	2.92	0	0.00	7	1.63
9	6	2.50	0	0.00	6	1.40
10	3	1.25	0	0.00	3	0.70
11	3	1.25	0	0.00	3	0.70
13	1	0.42	0	0.00	1	0.23
15	1	0.42	0	0.00	1	0.23
Total	240	100.00	190	100.00	430	100.00

In both the primary and permanent dentition, analysis of the dmft and DMFT components revealed that most teeth had caries and no filled teeth were identified (Table 2). In the primary dentition, the teeth most affected by dental caries were the mandibular left second molar (25.84%), mandibular right second molar (25.83%), and mandibular right molar (23.34%), whereas in the permanent dentition, the teeth most affected by dental caries were the mandibular right first molar (16.85%), mandibular left first molar (15.79%), and maxillary left first molar (7.37%).

**Table 2:** Absolute and percentage distribution of examined teeth, according to the components of the dmft and DMFT indexes in schoolchildren aged 5 and 12 years, respectively (Benguela, Angola, 2019)

Dentition	Healthy		Decayed		Missing		Filled		Total	
	n	%	n	%	n	%	n	%	n	%
Primary	3,988	88.37	502	11.12	23	0.51	0	0	4,513	100.00
Permanent	4,744	97.05	142	2.91	2	0.04	0	0	4,888	100.00
Total	8,732	92.88	644	6.85	25	0.27	0	0	9,401	100.00

Among 5-year-old students, it was observed that most individuals of both sexes had dental caries (Table 3). In contrast, most 12-year-old students of both sexes were free from dental caries (Table 4). Hence, there was no statistically significant association between gender and dental caries.

**Table 3:** Relationship between dental caries experience and sociodemographic characteristics in 5-year-old schoolchildren. Benguela, Angola, 2019

Variables	dmft = 0		dmft > 0		Total		*P* value	Odds ratio
	n	%	n	%	n	%		
Sex							0.90	
Male	48	42.11	66	57.89	114	100.00		–
Female	53	42.06	73	57.94	126	100.00		1.00
Total	101	42.08	139	57.92	240	100.00		
Housing location							0.01*	
Urban	36	46.75	41	53.25	77	100.00		–
Peri-urban	20	27.03	54	72.97	74	100.00		2.37
Rural	45	50.56	44	49.44	89	100.00		0.86
Total	101	42.08	139	57.92	240	100.00		
Educational level of the father/stepfather/guardian							0.81	
Primary school completed or lower	16	41.03	23	58.97	39	100.00		–
High school completed	10	50.00	10	50.00	20	100.00		0.70
Higher education completed	2	40.00	3	60.00	5	100.00		1.04
Did not know/did not answer	73	41.48	103	58.52	176	100.00		0.98
Total	101	42.08	139	57.92	240	100.00		
Educational level of the mother/stepmother/guardian							0.96	
Primary school completed or lower	19	40.43	28	59.57	47	100.00		–
High school completed	5	45.45	6	54.55	11	100.00		0.81
Higher education completed	2	40.00	3	60.00	5	100.00		1.02
Did not know/did not answer	75	42.37	102	57.63	177	100.00		0.92
Total	101	42.08	139	57.92	240	100.00		

The analysis of the relationship between the experience of dental caries and the location of schools revealed that there was a statistically significant association (P = 0.01) between the higher incidence of the disease and peri-urban location among the 5-year-old schoolchildren, with an odds ratio of 2.37 (Table 3). Among 12-year-old schoolchildren, the rural location had the highest proportion of individuals with dental caries experience, but there was no statistically significant association between these variables (Table 4). Furthermore, a large proportion of those in charge of the students had a low level of education. However, there was no association between this variable and the experience of dental caries, both in 5- (Table 3) and 12-year-old children (Table 4).

**Table 4:** Relationship between dental caries experience and sociodemographic characteristics in 12-year-old schoolchildren, Benguela, Angola, 2019

Variables	DMFT = 0		DMFT > 0		Total		*P* value	Odds ratio
	n	%	n	%	n	%		
Sex							0.19	
Male	70	67.31	34	32.69	104	100.00		–
Female	49	56.98	37	43.02	86	100.00		1.55
Total	119	62.63	71	37.37	190	100.00		
Housing location							0.70	
Urban	40	64.52	22	35.48	62	100.00		–
Peri-urban	19	67.86	9	32.14	28	100.00		0.86
Rural	60	60.00	40	40.00	100	100.00		1.21
Total	119	62.63	71	37.37	190	100.00		
Educational level of the father/stepfather/guardian							0.23	
Primary school completed or lower	17	56.67	13	43.33	30	100.00		–
High school completed	10	41.67	14	58.33	24	100.00		1.83
Higher education completed	11	68.75	5	31.25	16	100.00		0.59
Did not know/did not answer	81	67.50	39	32.50	120	100.00		0.63
Total	119	62.63	71	37.37	190	100.00		
Educational level of the mother/stepmother/guardian							0.20	
Primary school completed or lower	18	47.37	20	52.63	38	100.00		–
High school completed	9	64.29	5	35.71	14	100.00		0.50
Higher education completed	9	75.00	3	25.00	12	100.00		0.30
Did not know/did not answer	83	65.87	43	34.13	126	100.00		0.47
Total	119	62.63	71	37.37	190	100.00		

Access to dental services was a major concern in this population, with 51.67% (Table 5) and 62.63% (Table 6) of schoolchildren aged 5 and 12 years, respectively, having never been to a dental surgeon. There was no association between the time since the last dental appointment and the experience of dental caries.

**Table 5:** Relationship between dental caries experience, use of dental services, oral hygiene practices, and eating habits in 5-year-old schoolchildren, Benguela, Angola, 2019

Variables	dmft = 0		dmft > 0		Total		*P* value	Odds ratio
	n	%	n	%	n	%		
Time since last dental appointment							0.76	
Less than 1 year	22	39.29	34	60.71	56	100.00		–
More than 1 year	24	40.00	36	60.00	60	100.00		0.97
Never visited the dentist	55	44.35	69	55.65	124	100.00		0.81
Total	101	42.08	139	57.92	240	100.00		
Daily toothbrushing							0.88	
None	6	37.50	10	62.50	16	100.00		–
Once	47	43.52	61	56.48	108	100.00		0.78
Two or more times	48	41.38	68	58.62	116	100.00		0.85
Total	101	42.08	139	57.92	240	100.00		
Use of fluoridated toothpaste							0.72	
Yes	20	57.14	15	42.86	35	100.00		–
No	6	46.15	7	53.85	13	100.00		1.56
Does not know if the toothpaste contains fluoride	75	39.06	117	60.94	192	100.00		2.08
Total	101	42.08	139	57.92	240	100.00		
Frequency of fresh fruit intake							0.71	
Never	27	43.55	35	56.45	62	100.00		–
Once a week	39	39.00	61	61.00	100	100.00		1.21
Every day	35	44.87	43	55.13	78	100.00		0.95
Total	101	42.08	139	57.92	240	100.00		
Frequency of consumption of candies (cookies, cakes, pies, and the like)							0.90	
Never	4	36.36	7	63.64	11	100.00		–
Once a week	49	41.53	69	58.47	118	100.00		0.80
Every day	48	43.24	63	56.76	111	100.00		0.75
Total	101	42.08	139	57.92	240	100.00		
Frequency of intake of sugary drinks (milk, tea, juices, carbonated drinks, and the like)							0.19	
Never	10	38.46	16	61.54	26	100.00		–
Once a week	53	38.13	86	61.87	139	100.00		1.01
Every day	38	50.67	37	49.33	75	100.00		0.61
Total	101	42.08	139	57.92	240	100.00		

**Table 6:** Relationship between dental caries experience, use of dental services, oral hygiene practices, and eating habits in 12-year-old schoolchildren (Benguela, Angola, 2019)

Variable	DMFT = 0		DMFT > 0		Total		*P* value	Odds ratio
	n	%	n	%	n	%		
Time since last dental appointment							0.09	
Less than 1 year	12	48.00	13	52.00	25	100.00		–
More than 1 year	34	73.91	12	26.09	46	100.00		0.33
Never visited the dentist	73	61.34	46	38.66	119	100.00		0.58
Total	119	62.63	71	37.37	190	100.00		
Daily toothbrushing							0.08	
None	17	50.00	17	50.00	34	100.00		–
Once	101	66.45	51	33.55	152	100.00		0.51
Two or more times	1	25.00	3	75.00	4	100.00		3.00
Total	119	62.63	71	37.37	190	100.00		
Use of fluoridated toothpaste							0.94	
Yes	56	68.29	26	31.71	82	100.00		–
No	4	66.67	2	33.33	6	100.00		1.08
Does not know if the toothpaste contains fluoride	59	57.84	43	42.16	102	100.00		1.57
Total	119	62.63	71	37.37	190	100.00		
Frequency of fresh fruit intake							0.37	
Never	30	61.22	19	38.78	49	100.00		–
Once a week	78	65.55	41	34.45	119	100.00		0.83
Every day	11	50.00	11	50.00	22	100.00		1.58
Total	119	62.63	71	37.37	190	100.00		
Frequency of consumption of candies (cookies, cakes, pies, and the like)							0.20	
Never	2	50.00	2	50.00	4	100.00		–
Once a week	95	66.43	48	33.57	143	100.00		0.51
Every day	22	51.16	21	48.84	43	100.00		0.95
Total	119	62.63	71	37.37	190	100.00		
Frequency of intake of sugary drinks (milk, tea, juices, carbonated drinks, and the like)							0.28	
Never	10	83.33	2	16.67	12	100.00		–
Once a week	93	61.18	59	38.82	152	100.00		3.17
Every day	16	61.54	10	38.46	26	100.00		3.13
Total	119	62.63	71	37.37	190	100.00		

Although the majority of students brushed their teeth at least once daily, 6.67% of students aged 5 years and 17.89% of students aged 12 years did not. It was also established that none of the students used dental floss and that there was significant ignorance regarding the presence of fluoride in their toothpastes. However, there was no association between oral hygiene practices and dental caries.

Among 5-year-old students, the proportion of individuals who consumed candies (46.25%) and sugary drinks (31.25%) every day was 22.63% and 13.68%, respectively, which was higher than that observed among 12-year-old students. No association was found between the investigated eating habits and experience of dental caries.

## Discussion

In the present study on the epidemiological profile of dental caries in schoolchildren in the city of Benguela, a critical scenario of limited access to dental surgeons and total lack of treatment for teeth affected by dental caries was observed.

Studies evaluating the oral health status of children and adolescents in Uganda and Rwanda also reported limited access to dental services and a large number of cases with untreated oral diseases.^4,16^ This situation, which has been observed in several regions of the African continent, may be related to the shortage of dentists and the huge burden of dental diseases afflicting this population, suggesting the need to increase the number of oral health professionals to meet population demand.^27^ A recent study investigated the proportion of dentists in relation to the population by analysing the undergraduate and graduate dental courses in Angola and found that the country had only 701 registered professionals for a population of 30,175,553 inhabitants, that is, a ratio of 1:43,460.15 In addition to the incompatibility between the number of dentists and the population of Angola, it was found that no institution of higher education in the country offers postgraduate courses in dentistry and that there is no standardisation of pedagogical projects among the existing undergraduate courses.^15^ Furthermore, the paucity of dental professionals, materials, and equipment exacerbated the high demand for dental treatment, thereby increasing the costs of dental services and making financial barrier an important factor limiting access to dental services.^20,28^ This reinforces the importance of quantitative and qualitative training of dentists and specialists trained to provide adequate dental services to ensure access to oral health services for the population.^6,26^ In this study, no restored dental elements were found, either in the permanent or primary dentition, which could be a consequence of the limited availability and access to dental services.

In the present study, the analysis of dental elements demonstrated that the maxillary and mandibular molars were the most affected by tooth decay, which was similar to the results of a study conducted in young people in the Kingdom of Lesotho, South Africa.^12^ Based on this finding, it is reasonable to suggest that there is a significant lack in the use of preventive measures, such as the application of pit-and-fissure sealant, which could help reduce the prevalence and severity of dental caries and its sequelae in children and adolescents.^2,7^ The findings on oral hygiene practices also reflected the need to implement actions and strategies for oral health education and disease prevention, as none of the participants used dental floss and the majority did not know if the dentifrice they used was fluoridated. A study conducted in Nigeria concluded that promoting toothbrushing twice daily and reducing the frequency of consumption of sugary foods between meals constituted an effective public health strategy for oral health promotion and disease prevention.^10^ In the present study, although the majority of students brushed their teeth daily, a qualitative analysis of the control of dental biofilm was not performed, which could be a limitation of the study and should be evaluated in future research.

The greatest burden of oral disease worldwide is concentrated in low- and middle-income countries, which also face considerable social inequality.^18^ In this context, the present study found that the prevalence and severity of tooth decay in 5-year-old schoolchildren were higher among residents of the peripheral urban region than those in the central urban region. Similarly, a study on schoolchildren in the city of Maputo, Mozambique, showed that children from urban schools had a lower rate of dental caries than those in suburban schools.^14^ These findings are consistent with evidence demonstrating that the human resources and infrastructure required for oral health services in the West African region are concentrated in central urban areas, close to the higher-income population, while peripheral and rural communities have little or no resources.^11,21^ Such situations highlight existing inequalities in the accessibility, distribution, and use of oral health services, as well as in the prevalence of oral diseases, oral health knowledge, and oral hygiene practices.^21^ This may be related to the fact that people living in peripheral regions or far from central urban areas may have lower levels of education, lower health literacy, no health insurance coverage, or fewer financial resources to employ with dental care.^21^ Although studies suggest that the prevalence of dental caries in children has an inverse relationship with the level of education of parents, in this study, there was no association between these variables, which can be explained by the low level of education in almost the entire sample.^5,8^

In this study, a concerning situation was identified in relation to the occurrence of dental caries in the primary dentition of students in Benguela, emphasising the need for interventions that influence the most modifiable sociobehavioural and socioeconomic determinants, translating them into effective policies and programmes to prevent oral health diseases in childhood. Oral diseases represent an important public health problem that can negatively impact the quality of life of children and adults in both developed and developing countries.^3,19^ Childhood tooth decay is prevalent worldwide, but it has increased rapidly in recent years, especially in low- and middle-income countries, in parallel with changes in the population’s diet and lifestyle.^23^ In this study, it was found that 5-year-old students consumed candies and sugary drinks more frequently than 12-year-old students, which may explain the higher prevalence and severity of dental caries observed at this age, highlighting the need for dietary education strategies and the importance of implementing and maintaining proper eating habits, with foods with low cariogenic potential, for the prevention of dental caries and other oral diseases.^1,13^ As an aggravating factor, untreated caries lesions can cause pain and adversely affect the overall health, growth, development, and quality of life of children, their families, and the community.^23^

In this study, schoolchildren aged 5 and 12 years were examined; therefore, the findings cannot be extrapolated to the population of children of the aforementioned age indexes, but only to those inserted in the school context, which can be considered a limitation of the study. There is a scarcity of research data on oral health in the population of Benguela, resulting in insufficient scientific production and an inability to address all the existing and emerging challenges in healthcare.^18^ This highlights the importance of the findings of this study in planning oral health promotion strategies for the population of Benguela. Further research should be conducted to analyse the oral health condition of other age groups in the population of Benguela, especially adolescents and young adults, considering the possibility of an increase in the prevalence of dental caries due to the change in habits that may occur in this age group.

## Conclusion

The prevalence of dental caries in the permanent dentition of schoolchildren in Benguela is very low; however, in deciduous dentition, the situation is critical, especially among those living in peri-urban areas. The limited access to dental surgeons and the total lack of treatment for affected teeth demonstrate the need to establish a public policy to promote oral health. The use of dental floss as part of oral hygiene practices is not a reality in this population. The consumption of candies and sugary drinks, especially among 5-year-old children, deserves attention.
